# Case Report: A new patient expanding the clinical spectrum of *DOHH*-associated syndrome with increased nuchal translucency, cardiomyopathy, hypoparathyroidism and mild intellectual disability

**DOI:** 10.3389/fped.2026.1733592

**Published:** 2026-06-04

**Authors:** Elise Daire, Sabine Dirani, Karine Braun, Segolene Delmas Lanta, Hélène Cavé, Adeline Alice Bonnard, Benedicte Demeer

**Affiliations:** 1 Pediatric Cardiology Department, Amiens University Hospital, Amiens, France; 2 EA4666 Hematim, University of Picardie–Jules Verne, Amiens, France; 3 Department of Pediatrics, Amiens University Hospital, Amiens, France; 4 Department of Obstetrics, Gynaecology and Reproductive Medicine, Amiens University Hospital, Amiens, France; 5 Service de Génétique du Développement, Hôpital Robert Debré, AP-HP, Paris, France; 6 Université Paris Cité, Paris, France; 7 Genetic Department, CLAD, Amiens University Hospital, Amiens, France; 8 Chimere INSERM UA21, University of Picardie-Jules Verne, Institut Faire Face, Amiens, France

**Keywords:** cardiomyopathy, *DOHH*, hypocalcemia, increased nuchal translucency, neurodevelopmental disorders

## Abstract

Polyaminopathies are a family of rare genetic developmental disorders caused by pathogenic variants in genes involved in polyamine homeostasis and metabolism, and related biological pathways, including the hypusine biosynthesis pathway. DOHH is the enzyme catalyzing the last step of hypusine biosynthesis. *DOHH*-related disorders, caused by biallelic variants in the *DOHH* gene, were first described in 2022 in five patients exhibiting severe neurodevelopmental delay, congenital heart defects, and growth restriction. We report on a 10-year-old boy, the first child of consanguineous parents, whose clinical presentation began prenatally with increased nuchal translucency (7.5 mm), pericardial effusion and thickening of the right ventricular anterior wall. Postnatally, he developed dilated cardiomyopathy with left ventricular hypertrabeculation and hypoparathyroidism. Follow-up revealed delays in motor and cognitive milestones. Trio exome sequencing (ES) identified an inherited homozygous c.455C>T (p.Pro152Leu) pathogenic variant in *DOHH*. Review of the literature shows that our patient exhibits milder neurodevelopmental impairment and intellectual disability than previously described, and no growth restriction. Interestingly, this is the second report of increased prenatal nuchal translucency in *DOHH*-related disorders. As cardiomyopathy and hypoparathyroidism are being reported for the first time in this condition, additional cases are needed to corroborate or disprove this association. This case expands the phenotypic spectrum of *DOHH*-related disorders. It supports the diagnostic utility of exome sequencing in rare diseases, and highlights the importance of systematic bioinformatic reanalysis, long-term follow-up for accurate phenotypic characterization, and genetic counseling.

## Introduction

Deoxyhypusine hydroxylase (DOHH) is an enzyme characterized as a unique non-heme diiron monooxygenase with a superhelical structure, which is involved in the post-translational modification of the eukaryotic translation initiation factor 5A (eIF5A) (1). The eIF5A protein and its isoforms are the only known cellular proteins to contain hypusine, a unique amino acid derived from lysine (1, 2). This post-translational modification is essential for eIF5A activation and occurs through a two-step enzymatic process involving deoxyhypusine synthase (DHPS) and DOHH (1, 3). Hypusinated eIF5A is essential for translation and cell proliferation; consequently, the genes involved in this pathway - *EIF5A*, *DHPS*, and *DOHH* - are highly conserved across all eukaryotes (1).

Studies using knockout mouse models found an evolutionarily conserved role for the DOHH-mediated second step of hypusine synthesis in early embryonic development (4). Functional studies of fibroblasts derived from affected patients carrying biallelic *DOHH* variants, revealed an accumulation of deoxyhypusine-containing eIF5A [eIF5A(Dhp)] and a reduction in the hypusinated eIF5A, providing biochemical evidence for a deficiency in DOHH activity in cells (3). Moreover, the knockout of either the *EIF5A* gene or of the deoxyhypusine synthase gene (*DHPS*) caused early embryonic lethality in mice, indicating the essential nature of both eIF5A-1 and deoxyhypusine synthase in mammalian development (5).

Clinical descriptions of patients with variants affecting the hypusine biosynthesis pathway were first reported in 2019 in five patients with biallelic variants in *DHPS* (6) and subsequently in seven patients with *EIF5A* variants (7). More recently Ziegler et al. (2022), described five patients with biallelic pathogenic variants in *DOHH*, who presented with developmental abnormalities, including severe neurodevelopmental delay, congenital heart defects, and growth restriction (OMIM #620066) (3). The phenotypic spectrum associated with *DOHH*-related disorders remains to be fully delineated.

We report a novel clinical case of a patient with a *DOHH*-related syndrome, initially referred for increased nuchal translucency (NT), a common non-specific prenatal ultrasound finding. The emergence of cardiomyopathy, hypoparathyroidism, and mild intellectual disability during postnatal follow-up justified comprehensive genetic testing. Whole-exome sequencing revealed a homozygous missense variant in *DOHH*, leading to the diagnosis of this rare syndrome.

## Case description

We report a 10-year-old boy, the first child of consanguineous parents, born at 38 weeks of gestation, with normal birth metrics: weight 3,640 g (+0.3SD), length 49 cm (−1.1SD), head circumference 36.4 cm (+1SD), and Apgar scores of 10,10 at 1 and 5 min, respectively (8).

During the first trimester of pregnancy, an increased NT measurement of 7.5 mm was observed. Due to technical difficulties during sampling, only common aneuploidies (trisomies 21, 18 and 13), could be excluded on chorionic villi sampling. A complementary array-CGH, which was the standard option available in France at that time, was proposed. As this required an additional amniocentesis, the couple decided not to take the associated risks of a further invasive procedure. In the second trimester, fetal echocardiography revealed a mild pericardial effusion with apparent thickening of the anterior wall of the right ventricle and normal fetal growth.

**Figure 1:**
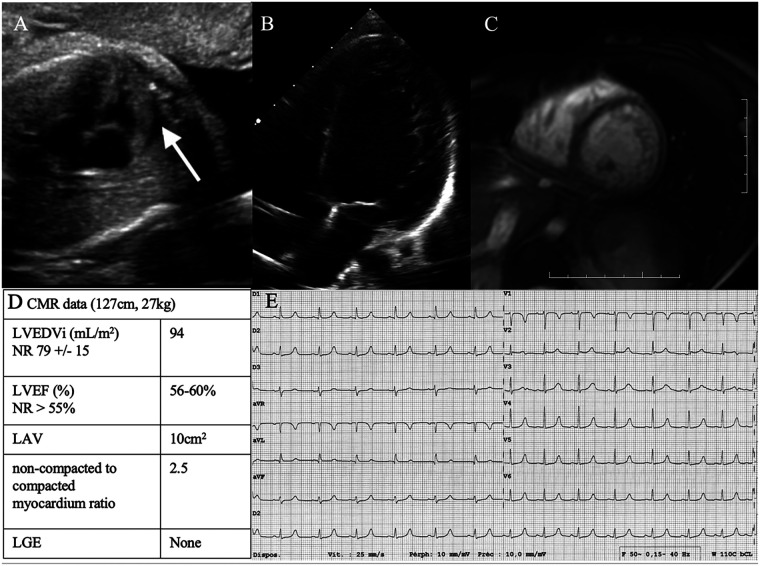
Fetal echocardiogram, transthoracic echocardiography, electrocardiogram, and cardiac magnetic resonance (CMR) data. **(A)** Fetal echocardiogram apical four-chamber view demonstrating a pericardial effusion (white arrow), **(B)** Four-chamber echocardiogram at end-diastole at last assessment, **(C)** CMR short axis view at end-diastole, **(D)** CMR data, **(E)** Electrocardiogram at last assessment. CMR, cardiac magnetic resonance imaging; LAV, left atrial volume; LGE, late gadolinium enhancement; LVEDVi, indexed left ventricular end-diastolic volume; LVEF, left ventricular ejection fraction; NR, normal range.

Postnatal cardiac assessment identified left ventricular (LV) hypertrabeculation without ventricular dilatation, preserved ventricular function, and normal coronary implantation. No pericardial effusion was observed; however, a restrictive apical ventricular septal defect was detected. Parental echocardiography evaluations were normal. Follow-up showed diffuse LV hypertrabeculation with mild LV dilatation, but preserved function and no myocardial fibrosis as confirmed on cardiac magnetic resonance (CMR), performed at six years of age (Figures 1A–E). The ventricular septal defect had spontaneously closed at three years of age. At the most recent follow-up, at ten years of age, the patient remained asymptomatic, with a stable cardiac phenotype and without any arrhythmic events.

At three years of age, hypocalcemia was incidentally detected on a routine blood test. An analysis of phosphocalcic metabolism revealed isolated hypoparathyroidism, requiring calcium and vitamin D supplementation. Parental biological screening for familial hypoparathyroidism was unremarkable.

The patient presented with a mild neurodevelopmental delay, sitting at 9 months and walking at 18 months. At six years, a neuropsychological assessment revealed fine motor and visuospatial deficits, while a speech-language evaluation identified developmental dysphasia. Psychometric testing (WISC-V) confirmed a mild intellectual disability (FSIQ 50–61). Since the age of seven, he has attended a specialized educational institution.

The patient presented with distinctive craniofacial features, including downward-slanting palpebral fissures, malar hypoplasia, and a thin upper lip with smooth philtrum. He had blue sclerae and bright vivid blue irises. An ophthalmological fundus examination showed bilateral retinal hypopigmentation. Skin examination revealed dryness of the extremities, particularly on the hands, but no cutaneous or limb abnormalities were noted. Audiometric evaluation showed normal hearing. Immunologic blood tests did not reveal any immunodeficiency. At his last evaluation at 10 years of age, his growth was in the normal range.

Given the association of prenatal increased NT, cardiomyopathy, ophthalmological findings and distinctive facial features, postnatal investigations included an array-CGH and a dedicated panel for Noonan syndrome and related disorders, both of which were inconclusive. Trio exome sequencing (ES) was then performed identifying an inherited homozygous c.455C>T; p.(Pro152Leu) variant in the *DOHH* gene (NM_001145165.1), classified as likely pathogenic according to ACMG criteria (9). This variant has previously been reported in a compound heterozygous state, and functionally studied, with evidence supporting its deleterious impact on DOHH activity and pathogenicity (3). Reanalysis of ES data after the onset of cardiomyopathy and hypoparathyroidism did not identify any additional pathogenic variants in genes known to be associated with these conditions.

## Discussion

We report on a new patient with a *DOHH*-related disorder - also known as neurodevelopmental disorder with microcephaly, cerebral atrophy, and visual impairment NEDMVIC (OMIM #620066) - a clinical entity first described in 2022 in five patients (3). The homozygous c.455C>T;p.(Pro152Leu) substitution in *DOHH*, identified in our patient, affects a highly conserved amino acid residue, and is predicted to alter the enzyme's structure and/or activity (3).

Interestingly, our patient presents with a milder neurodevelopmental delay than the five patients previously described, who exhibited severe neurological impairment, including hypotonia, and marked delays in motor milestones (Table 1). Moreover, our patient does not present with microcephaly, focal neurological signs, and visual impairment, as reported in the initial description of “NEDMVIC”.

**Table 1:** Literature review of *DOHH*, *DHPS* and *EIF5A* cardinal signs adapted from (3, 6, 7).

Reference	Gene	Prenatal features		Postnatal features					
		Increased NT	IUGR or pre-eclampsia	Cardiac involvement	Microcephaly (−3 to −6 SD)	Hypotonia	Development delay or intellectual disability	Seizures	Hypoparathyroidism
Reported patient (*n* = 1)	*DOHH*	1	0	CM	0	0	Mild	0	1
Ziegler et al., 2022 (2) (*n* = 5)	*DOHH*	1	0	3 CHD	5	5	5 Severe	3	0
Ganapathi et al., 2019 (4) (*n* = 5)	*DHPS*	2	2	0	2	4	5 Moderate to severe	5	0
Faundes et al., 2021 (5) (*n* = 7)	*EIF5A*	0	4	4 CHD	5	3	7 Mild to moderate	0	0

CHD, cardiac heart defect; CM, cardiomyopathy; IUGR, intrauterine growth restriction; NT, nuchal translucency.

In addition to these neurological features, the broader clinical picture of our patient began prenatally with an increased NT, a feature previously reported in association with chylothorax in one individual (3). Postnatally, his growth parameters have remained normal, differing from the growth restriction reported in the original cohort (length −1.8 to −3.28 SD and weight −2 to −4.86 SD). Moreover, while cardiac involvement in most previously described patients (3/5) primarily consisted of congenital heart defects and conduction abnormalities, our patient presented with cardiomyopathy (Table 1) (3). Given the onset of hypoparathyroidism in our patient—a feature not previously reported—we recommend biochemical screening in affected individuals. Since our patient is now ten years old, this case provides a valuable longitudinal perspective on the evolution of the phenotype. To date, the oldest reported individual was 17 years of age at the time of publication; however, mortality in the original cohort was high, with two out of five patients dying (one at 25 months and the other at 15 years of age). Altogether, this report highlights the broad clinical variability of *DOHH*-related disorders. Such phenotypic differences raise questions about the role of additional genetic or environmental modifiers.

**Figure 2:**
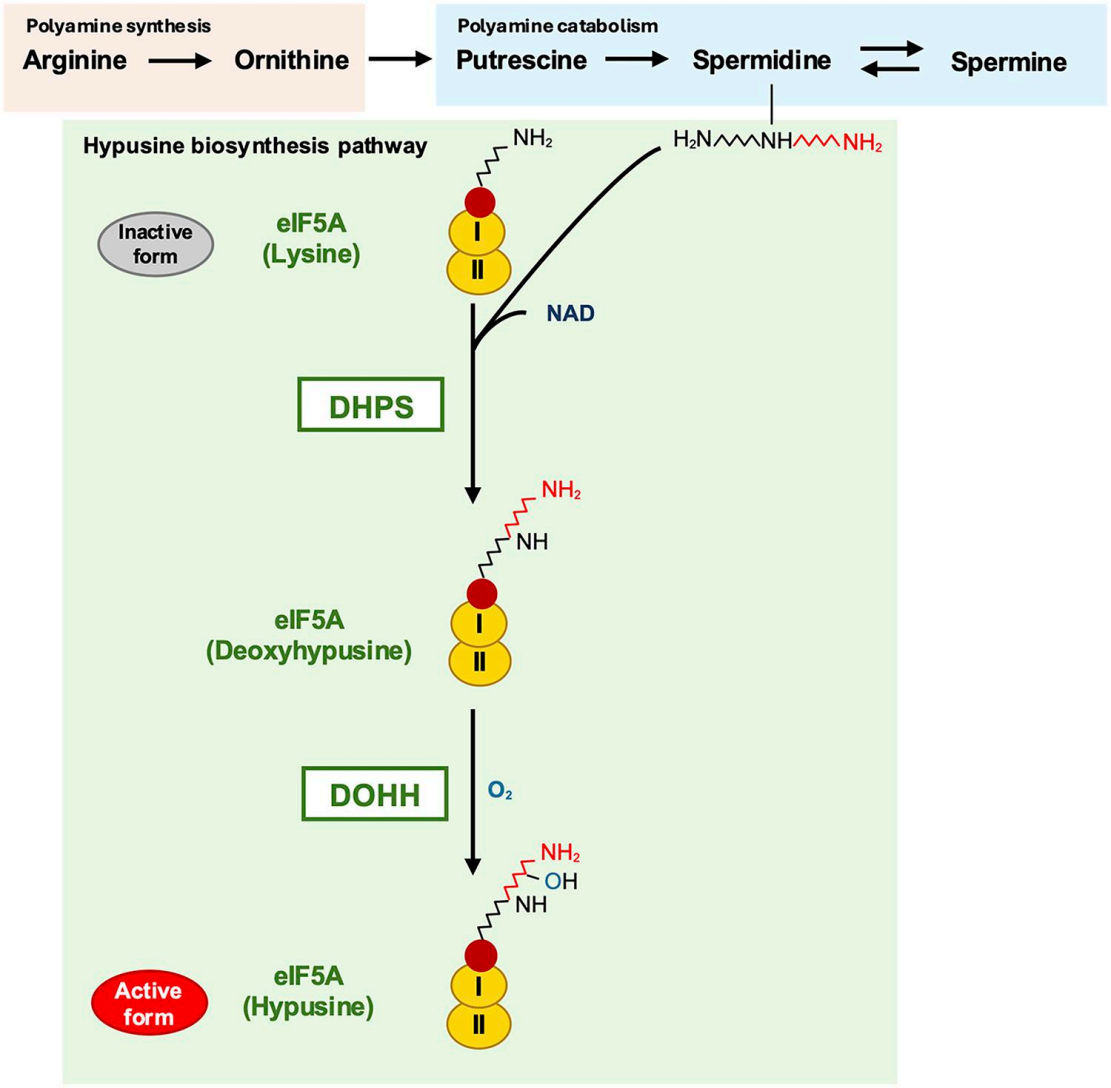
Pathways of polyamine biosynthesis and hypusine modification in eIF5A. Arginine is converted to ornithine, which is subsequently converted into putrescine, and further into spermidine and spermine. Post-translational hypusine modification of eIF5A occurs in two enzymatic steps catalyzed by deoxyhypusine synthase (DHPS) and deoxyhypusine hydroxylase (DOHH), generating the active, hypusinated form of eIF5A. The N-terminal domain (I) and C-terminal domain (II) of eIF5A are represented by yellow circles, and the exposed highly conserved loop containing the lysine/hypusine modification site is represented by a red circle.

DOHH acts as the second enzyme in the hypusine biosynthesis pathway, which also involves DHPS and eIF5A (Figure 2). Recently, Ziegler et al. proposed classifying the diseases related to this biosynthesis pathway under the term “*EIF5A* and hypusination-related disorders” (3, 6, 7). The cardinal clinical features shared among these conditions include microcephaly, developmental delay and intellectual disability, which are particularly severe in individuals with *DOHH* and *DHPS* variants (Table 1). Cardiac involvement has been observed in individuals with both *DOHH* and *EIF5A* variants, consisting exclusively of congenital heart diseases. Notably, hypoparathyroidism has never been reported in this spectrum.

Furthermore, as reviewed by Van Sickle et al. (2025), the hypusine biosynthesis pathway is adjacent and tightly connected to the polyamine pathway (2). To date, five known polyaminopathies have been described: *EIF5A*-related disorder also known as Faundes–Banka syndrome; *DHPS* deficiency; *DOHH*-related disorder; Snyder–Robinson syndrome; and Bachmann–Bupp syndrome. The latter two, Snyder–Robinson and Bachmann–Bupp syndromes, are caused by pathogenic variants in the spermine synthase (*SMS*) and ornithine decarboxylase 1 (*ODC1*) genes, respectively, whose encoded enzymes are involved in polyamine synthesis and catabolism. So far, apart from nonspecific signs such as global developmental delay and hypotonia, few distinctive signs have been associated with this broader group of polyaminopathies. Additional reports are needed to better delineate the clinical and molecular spectrum of these newly described disorders. Given the biological importance of the polyamine pathways and the clinical consequences of its disruption, it is reasonable to speculate that variants in other genes encoding components of this pathway may underlie additional, as yet undescribed, disorders.

During the prenatal period, our patient presented with an increased NT (NT > 95th percentile in the first trimester), a common indication for referral to a fetal medicine unit. Although strongly associated with chromosomal anomalies, an isolated increased NT poses a major genetic counseling challenge due to prognostic uncertainty once standard chromosomal causes are ruled out. Interestingly, this is the second report of an increased NT in *DOHH*-related disorders (3). Prenatal signs associated with eIF5A and hypusination-related disorders are commonly reported; a review of literature shows increased NT (*n* = 4/18), congenital heart diseases (*n* = 7/18), intrauterine growth restriction or pre-eclampsia (*n* = 6/18) (Table 1). This contrasts with polyhydramnios reported in Bachmann–Bupp syndrome (*n* = 7/12) and the paucity of prenatal findings in Snyder-Robinson syndrome (*n* = 5/33).

With rapid advances in sequencing technologies and genetic knowledge, most novel molecular diagnoses arise from the discovery of new disease genes, the recognition of additional clinical phenotypes, and the bioinformatic reanalysis of sequencing data, including upgraded variant classifications in known disease genes (10). This case supports the routine use of exome sequencing and potentially whole genome sequencing in the future, as a diagnostic test in rare diseases. Although not recommended as a routine prenatal diagnostic test, ES has been increasingly adopted in the clinical prenatal setting, especially when multiple fetal anomalies are present. Experience has been gained from postnatal genetic diagnosis. Several issues still need to be addressed for data interpretation and clinical reporting with the advent and application of such sequencing to prenatal diagnostics. First, prenatal disease phenotypes are less extensively characterized than postnatal ones, limiting data interpretation. Second, as with postnatal testing, systematic bioinformatic reanalysis of fetal sequencing data and careful reporting are crucial for accurate clinical diagnosis. Third, incidental findings are inevitable, including postnatally actionable variants, disorders without prenatal manifestations and misattributed parentage. The conditions under which they are reported should be clearly defined in accordance with parental preferences and informed consent.

## Conclusion

This case reinforces the role of *DOHH* in developmental disorders and highlights its potential association with cardiomyopathy and endocrine dysfunction. The clinical variability, including neurodevelopmental outcomes, poses challenges for genetic counseling, underlining the need for detailed phenotypic characterization. Further studies are needed to better characterize the clinical implications of *DOHH* variants, especially with long-term follow-up.

In the context of rare diseases, reporting clinical descriptions and the long-term follow-up of patients with newly identified disorders is essential for medical management, sequencing data interpretation, genetic counseling, and the better prenatal and postnatal characterization of these diseases.

## Data Availability

Variants reported in this study have been submitted to ClinVar (https://www.ncbi.nlm.nih.gov/clinvar/) and are publicly accessible under accession numbers [SCV006557881.1].
